# Clinical Lecture on the Significance of Uterine and Vaginal Discharges

**Published:** 1872-05

**Authors:** Robert Barnes

**Affiliations:** Obstetric Physician to St. Thomas’s Hospital, etc., etc.


*Clinical Lecture on the Significance of Uterine and Vaginal Discharges.* By Robert Barnes, M.D., Obstetric Physician to St. Thomas's Hospital, etc., etc.

*Discharges of Air.*—Air may get into the vagina, if not into the uterus, in the non-pregnant state. In the normal condition the walls of the vagina are maintained in perfect contact, and no air, or probably very little, is admitted. But where the parts are greatly relaxed, the vulva open as when the perineum is torn, the lower part of the vagina is no doubt exposed to the contact of air. But the very condition of patency prevents the retention of the air to such a degree as to lead to its escape in perceptible volume. Air also penetrates where too large a pessary is worn, which keeps the vaginal walls apart. But under peculiar circumstances air enters in large quantity, to be expelled with noise. Dr. George Harley details* a curious case in which he carried out decisive experiments to prove the correctness of the diagnosis. A pluripara frequently expelled air from the vagina with a loud noise. It was ascertained that no connection existed between the rectum and vagina. Dr. Harley took a full-sized male catheter, to which was attached a long India-rubber tube with a stopcock at the other end. The catheter was introduced into the uterus, the end of the tube with the stopcock being placed in a tumblerful of water. No air escaped when the instrument was in this position; but, on placing the open end of the catheter in the vagina, an instantaneous discharge of gas took place. The water was found to be sucked up through the tube into the vagina. It was found that the vagina sucked in and expelled the air by spasmodic action. It was further observed that the abdominal muscles assisted in the suction process. The uterus was completely retroverted. This displacement being remedied, and the patient's health improved by tonics, a cure ensued.

If you observe the vagina when the duck-bill speculum is applied, you will see movements of rise and fall under the influence of the rise and fall of the diaphragm. Dr. Adolph Rasch has investigated† the phenomena with great care. He says, if a multipara whose genitals are normal be placed on her back, with the thighs flexed and abducted, and the vaginal orifice closed, movements caused by respiration are seen, but no air enters. In the lateral position the same thing is observed even if the vagina is lax, and even when the perineum is ruptured. When the patient is placed in the prone position, or on all-fours, if the vulva be open, air will enter, because the intestines falling downwards by gravity cause a vacuum. Under this condition violent exertion may expel air, giving rise to vaginal flatus. If the abdomen be supported by the hands or by a bandage, no air enters.

There are several interesting applications of this knowledge. It teaches that the best position after labor, if not during labor also, is the dorsal; that the same position is also best in the case of pelvic abscess or hæmatocele discharging into the vagina; and that we must carefully consider this respiratory rise and fall of the vagina when selecting pessaries. It is by turning to account this

* Obstetrical Transactions, 1863.

† Ibid., 1870.

action that we derive the greatest advantage from the spoon or Sims's speculum. The blade drawing the perineum well back, whilst the semi-prone position of the patient favors the falling forwards of the abdominal viscera, air fills the vagina, counteracts the effect of inspiration, and thus enables us to get a good view of the os uteri. The same position also greatly aids our efforts at reducing inversion of the uterus, and in replacing a prolapsed umbilical cord.

In most operations, however, upon the uterus and vagina, where it is of importance to bring the uterus as low down near the vulva as possible, the dorsal position, by bringing the force of gravity to counteract the respiratory rise of the uterus, which can further be greatly aided by direct pressure by an assistant's hand above the symphysis pubis, is the best.

If we were to follow out in detail all the indications supplied by the examination of the discharges, we should be led through almost the entire range of uterine and vaginal pathology. This, in fact, is simply the converse proposition to one already stated—namely, that almost every uterine or vaginal disease is attended by discharge. But to adopt the discharges as our point of departure, from which to proceed to study and classify the diseases of which they are consequences, would be a most intricate course, and not the most orderly or profitable. I will therefore not pursue this method further than to give one instance of how we may apply it to clinical analysis. We will at our next meeting take hæmorrhage, and sketch what its purport is.

*The Watery Discharges.*—When these occur, you must, first of all, determine the presence or absence of pregnancy. It is no uncommon thing that discharges of water, more or less profuse, take place in pregnant women. This is the hydrorrhœa gravidarum. Gushes of water, quite clear, may occur at almost any time during pregnancy; but they are more frequent in the latter months, and especially in the last month. Happening at this time, they are commonly taken as an indication of commencing labor, and many are the false alarms which patient and doctor have to suffer from this cause. "The waters have broke," says the nurse. You go, as in duty bound, and find probably the os uteri closed, nothing resembling active labor-pains. What are you to do? If you wait for labor, you may wait for a week, or two or three weeks. If, on examination by ballottement, you find the child still floats in the uterus, the os uteri not open, and no active pains, you may go home and wait in peace for another summons.

What is the source and nature of this hydrorrhœa gravidarum? Several theories have been expounded. The character of the fluid differs in some respects from that of liquor amnii. It is odorless, and resembles blood-serum, or the serous fluid effused in the peritoneal sac. Ruysch and Rœderer thought it came from ruptures of lymphatic vessels, or of hydatids of the uterus Bohmer thought it escaped from a second abortive ovum; Delamotte and Cruveilhier that it came from a cyst near the ovum; Deleurye, Puzos, Naegele and Dubois that it came from the inner surface of the uterus, being secreted externally to the ovum. Dubois says it is the result of loosening of the membranes from the uterus when the vessels pour out serum. Hegar says the source is the uterine glands of the decidua. Thus, he describes* the glands of the mucous membrane as being found in the decidua at the sixth month of gestation, and argues that their sudden disappearance in the subsequent months is improbable. In a case of hydrorrhœa he found in the decidua vera, at the beginning of the eighth month, an enormously developed glandular body. At the bottom of this morbid growth was a general hypertrophic condition of the decidua and its glands. These gave out the excessive secretions.

In a case related by Dr. Graef,† repeated discharges took place, and the fœtus was expelled at the end of six months. The membranes were very delicate, and openings were found in them. In this case, it is probable that the fluid was true liquor amnii. In another case the patient suffered, during the last three months, from repeated watery discharges; the uterus rising and falling with the gathering and escape of the fluid. The membranes were found without rent. Graef regarded this as a case of catarrhal hydrorrhœa.

I believe there are various sources. In some cases the fluid is liquor amnii. This may come either from rupture of the membranes; from rapid transudation under pressure; from rapid formation and accumulation of liquor amnii in the amnion; or from the bursting of a cyst formed between the amnion and chorion, or between two layers of chorion, the proper amniotic sac remaining intact. In the majority of cases, however, the fluid is not amniotic; for when once the amniotic sac has fairly ruptured, labor is not far off. It is, then, the result of a rapid secretion from the uterine glands, or from the cervical cavity. In the early months, whilst there is still a free space between the decidua vera and the decidua reflexa, there is a large area of developed glandular surfaces.

I have observed a *puerperal form of hydrorrhœa* Thus, watery discharges may continue for a month or longer beyond the proper lochial flow. Generally in these cases the water is dirty, discolored, occasionally stained with blood, and offensive. The most common cause I have found to be the retention of a portion of placenta, or of clots in the uterus; but a polypus may produce

* Monatsschrift fur Geburtskunde, 1863.

† Jenaische Zeitschrift, 1865.

like results. The watery discharges alternate, but not always, with discharges of blood. The fluid may, under certain conditions, collect in considerable quantity in the uterus, so that the organ becomes greatly distended before the collection is expelled in a gush.

Sometimes watery fluid is mingled with air, constituting *physo-hydrometra*. This is also a puerperal or post-puerperal condition, and is commonly the result of retention of some portion of placenta or membranes, and the admission of air into the uterine cavity. If an examination is made when the uterus is relaxed after labor, especially if the hand be introduced into the uterus, the vaginal walls are separated from their usual contact, and a channel is formed along which air easily enters. Merely turning on the side, or a little more prone, will often, by favoring the fall of the uterus forwards, produce a vacuum into which air will rush. This is one reason, amongst others, why I am unable to approve of the abolition of the old-fashioned binder, which some people would condemn for no better reason that I can see than because it is old-fashioned. After labor, especially in pluriparæ, the abdominal walls are so relaxed that they can give no support to the uterus. The binder does temporary duty for the inert abdominal walls.

The history of physo-hydrometra is, I believe, this: A portion of placenta, membranes, or clots remains in the cavity of the uterus after labor; some air gets in as I have described; decomposition ensues, and the gases of putrefaction are added to the air from without, whilst the os uteri is occluded by the placenta or blood-mass falling over it. When this occurs, there is invariably hectic or irritative fever; peritonitis and septicæmia commonly attend; great abdominal pain; the enlarged, distended uterus can be mapped out rising as high as, or higher than, the umbilicus; and resonance is made out on percussion.

One condition, the result of impregnation, often leads to copious and repeated discharges of watery fluid; the *hydatidiform degeneration of the chorion*. In this case the ordinary signs of pregnancy may not be present, and even the patient herself may not think she is pregnant. There is, however, always evidence of enlargement of the uterus, and generally great pelvic distress. The water escapes in gushes at uncertain times; it is often tinged with blood, resembling red-currant water; it has not the offensive odor belonging to the watery discharges of cancer; sometimes, but not often until late in the progress of the case, cysts will be found swimming in the water; it is generally expelled with painful uterine contractions.

In a case we recently had in Adelaide ward, the nature of the disease was not at first suspected. There was some abdominal enlargement, retention of urine requiring the catheter, and most distressing pelvic pain with irritative fever. The os uteri was found high up above the symphysis pubis, whilst behind it the pelvic cavity was filled with a large, rounded, firm mass, taken to be either the retroverted gravid womb, or a fibroid tumor. One day a large quantity of water, blood, and a mass of chorion-cysts were expelled. We had, in fact, the condition of retroverted gravid womb complicated with hydatidiform or cystic degeneration of the chorion.

Apart from pregnancy, watery discharges are often of grave significance. During and after the climacteric period, the most frequent cause is some form of malignant disease, especially the so-called cauliflower excrescence of the uterus. In this case other symptoms will probably point to the seat and nature of the disease. The fluid discharged is seldom clear; it is generally turbid, dirty, often tinged with blood, resembling water in which flesh has macerated; it contains shreds or flocculi of solid matter, the proceeds of superficial erosion or necrosis of the surface of the diseased growth, and is almost always of a peculiar offensive odor. It often alternates with hæmorrhage. Local exploration will place the nature of the case beyond doubt. Another form of malignant disease giving rise to watery discharges is the "oozing excrescence of the labia."

But we must remember that similar discharges may take place from polypus or inversion of the uterus. Hence we have another example of the wisdom of not pronouncing a diagnosis until we have made an internal examination. Water may escape in large quantity from the rupture or perforation of an ovarian cyst into the vagina. In such a case, the rapid concurrent diminution of the abdominal tumor will lead to the right conclusion.—*London Lancet.*

